# Association between maternal literacy and child vaccination in Ethiopia and southeastern India and the moderating role of health workers: a multilevel regression analysis of the Young Lives study

**DOI:** 10.1080/16549716.2019.1581467

**Published:** 2019-04-08

**Authors:** Hwa-Young Lee, Juhwan Oh, Jongho Heo, Atakelti Abraha, Jessica M. Perkins, Jong-Koo Lee, Thi Giang Huong Tran, S. V. Subramanian

**Affiliations:** a JW LEE Center for Global Medicine, Seoul National University College of Medicine, Seoul, Republic of Korea; b Takemi Program in International Health, Department of Global Health and Population, Harvard T.H. Chan School of Public Health, Boston, MA, USA; c Ethiopian Health Insurance Agency, Ministry of Health, Addis Ababa, Ethiopia; d Department of Human and Organizational Development, Peabody College, Vanderbilt University, Nashville, TN, USA; e Vanderbilt Institute of Global Health, Vanderbilt University Medical Center, Nashville, TN, USA; f Department of Family Medicine, Seoul National University College of Medicine, Republic of Korea; g International Cooperation Department of Vietnam, Ministry of Health, Hanoi, Vietnam; h Harvard Center for Population and Development Studies, Harvard T.H. Chan School of Public Health, Boston, MA, USA; i Department of Social and Behavioral Sciences, Harvard T.H. Chan School of Public Health, Boston, MA, USA

**Keywords:** Vaccination, maternal literacy, health center, health workers, multilevel regression

## Abstract

**Background**: Child vaccination coverage in low- and middle-income countries is still far from complete, mainly among marginalized people such as children with illiterate mothers.

**Objective**: This study aims to examine the association between maternal literacy and immunization status of children in Ethiopia and southeastern India (Andhra Pradesh and Telangana) and test whether state-run health centers and community health workers moderate that association.

**Methods**: This study is based on cross-sectional data from samples of children in Ethiopia and India, collected as part of round 2 within the Young Lives study (2006). Multilevel logistic regression was conducted to estimate the association between maternal literacy and the completion of four kinds of child vaccinations. We further tested for cross-level interactions between state-run health centers or community health workers and maternal literacy. Estimates were adjusted for several individual- and household-level demographic and socioeconomic factors.

**Results**: Literate mothers were more likely to complete all four kinds of vaccinations for their children compared to illiterate mothers in Ethiopia (Odds Ratio (OR)=4.84, Confidence Interval (CI)=1.75-13.36). Presence of a health center was positively associated with completed vaccinations in India only (OR = 6.60, CI = 1.57–27.70). A cross-level interaction between community health workers and maternal literacy on the vaccination completion status of children was significant in Ethiopia only (OR = 0.29, CI = 0.09–0.96).

**Conclusions**: Our findings suggest that increased availability of community health workers may reduce the child vaccination gap for illiterate mothers, depending on the country context.

## Background

Communicable diseases are the main causes of child death throughout low- and middle-income countries (LMICs) even though many of them can be easily prevented by vaccination [1]. Given that most vaccinations are not expensive [2,3], proper vaccination can be one of the most cost-effective public health initiatives to decrease the global burden of vaccine-preventable diseases [4]. Accordingly, the World Health Organization (WHO) introduced the Expanded Program on Vaccination (EPI) in 1974 to target six diseases first (diphtheria, whooping cough, tetanus, measles, poliomyelitis and tuberculosis). Hepatitis B and Haemophilus influenzae type b (Hib) were included later [5]. Although EPI has made substantial contributions to increasing vaccination coverage in some LMICs [6], an estimated 18.7 million infants in LMICs were still missing basic vaccinations as of 2014. Furthermore, the coverage rate of most essential vaccinations in most African countries was less than 80% in 2014.

Child vaccination is of great importance in India where one third of the annual vaccine-preventable childhood deaths occur [7]. Despite almost three decades of a universal immunization program, about 39% of children aged 12–23 months did not receive a schedule of vaccination in 2014. Thus, about one in three children born every year do not receive protection, placing them at the highest risk of mortality and morbidity [7,8].

In 2014, Ethiopia recorded vaccination coverage rates of 78%, 84%, and 81% for bacillus Calmette–Guérin (BCG), three doses of diphtheria, tetanus, and pertussis vaccine (DPT3), and measles-containing vaccines (MCV), respectively, which were far below global averages (91.3%, 90.4%, and 89.5%, respectively) [9]. Although Ethiopia’s immunization program has improved in recent years [10], substantial coverage disparities across subgroups remain [11﻿–13]. Literacy is a critical factor for accessing vaccination service [14].

Several recent studies on child vaccination in LMICs have found a significant association between parental or caregiver literacy and child vaccination [15–19]. Caregiver illiteracy adversely affects child vaccination status via the capacity to obtain, process and understand basic information on the benefits and risks associated with child vaccination, which, in turn, lead to poor adherence to the recommended vaccination schedule [20–22]. Community health centers and health workers, called Anganwadi workers (AWW) in India or health extension workers in Ethiopia, may play an important role in improving vaccination coverage for marginalized children with illiterate caregivers because such workers are often responsible for managing the vaccination process within their community [23]. For example, they rule out contraindications, record vaccination status, check vaccine transportation and storage to ensure efficacy, and inform parents about vaccination benefits and risks [24,25].

Associations between parental literacy and child vaccination status, and between community factors such as the presence of community health centers or health workers and vaccination coverage, have been found in previous studies [23,26]. However, the role of these community factors as a moderator of the association between maternal literacy and child vaccination status remains unknown. Thus, the present study aims to test two hypotheses: (a) whether there is a positive association between maternal literacy and child vaccination, and (b) the extent to which the association between maternal literacy and child vaccination status is moderated by the presence of state-run health centers and community health workers.

## Methods

### Study design

Data were obtained from an international and longitudinal survey named the ‘Young Lives Study’. The Young Lives study is comprised of two cohort groups, the first of which is a ‘younger cohort’ including 1999 and 2011 children aged between 6 and 18 months in Ethiopia and India, respectively. The second group, labeled the ‘older cohort’, included 1000 and 1008 children aged 7 to 8 years in Ethiopia and India, respectively, at the time of recruitment in 2002.

The first round of data from the younger cohort was obtained in 2002 (age of cohort: 6 to 18 months), the second round in 2006–2007 (age of cohort: 4 to 5 years), and the third round in 2009–2010 (age of cohort: 7 to 8 years). Our study mainly used the round 2 data set from the younger cohort because it included information on basic vaccinations which should have been completed when the child reached the age of 4 to 5 years. Only sibling status was extracted from the round 1 data set as that information was only collected during the first round.

### Participants and sampling

The Young Lives study employed a clustered sampling strategy with a semi-purposive sampling of 20 sentinel sites, with oversampling of sites covering poor areas rather than nationally representative sampling in each country, because the aim of the study was to assess the causes and consequences of childhood poverty [27]. In India, sentinel sites were chosen only within the states of Andhra Pradesh and Telangana, while sites were selected nationwide in Ethiopia. The sentinel sites include both urban and rural areas, representing a range of regions, policy contexts, and living conditions. Within each sentinel site, all households containing children in the target age groups were identified and listed, from which 150 (100 for the younger cohort and up to 50 for the older cohort) were randomly selected [27–29]. A comparison was made between the study samples and nationally representative samples using data from the Welfare Monitoring Survey 2000 for Ethiopia and data from the Demographic and Health Survey 1998/99 for India. Comparison of several living standard indicators showed that the samples in the Young Lives Study were slightly better off, which might be partly explained by a substantial decrease in national poverty rates over the gap between the survey year of the Young Lives Study and nationally representative samples. More information on the sampling strategies in each country can be found elsewhere [30,31].

Households that refused to participate (representing less than 2% of the selected households) were replaced with other households from the list. The response rate was above 90% in both countries. Attrition rates were notably low compared to other longitudinal studies in similar contexts, ranging from 0.50 to 3.52%, and were similar across countries [32]. Data were collected from the child’s main caregiver, which was either the child’s mother (mainly) or father (only minimally), using a standardized, interviewer-administered questionnaire. All interviewers received training based on common guidelines. After excluding cases with missing information or ‘don’t know’ responses for the outcome variable and any of the explanatory variables, 1157 children in 22 communities in Ethiopia and 1455 children in 75 communities in India comprised the final analytic samples for this study.

### Outcome measure

A binary variable indicated whether the respondent’s child had completed four kinds of vaccinations including BCG, MCV, DTP3, and polio. We did not include Hib because Hib was introduced in Ethiopia in 2006 and in India in 2009, which is much later than our survey period. Answers to these questions were obtained from the child’s vaccination card if it was available. Otherwise, the answer was based on the respondent’s recall (yes/no) for each vaccination.

### Explanatory measures and covariates

Respondents were asked whether the mother and father of the index child could read and understand a letter or newspaper in their own language, which was Telugu in India, and the most commonly used language in that locality in Ethiopia. Each parent was assessed as illiterate if they could not read and understand it at all or as literate if they could read and understand it easily or with difficulty.

Data on state-run health centers and community health workers were collected by asking key informants in the community, such as a community leader or village representative, whether community health workers were present and delivered their services in the locality and whether the state-run health centers were available 4 years prior when the index child received a series of vaccinations. The definition of community health workers did not include social workers or mental health workers. State-run health centers did not include health posts.

Other explanatory variables used in this study were maternal age, child’s gender, child sibling status, household wealth status, type of residence, and region. Maternal education level was not included due to concerns about the high probability of collinearity with the literacy variable and the over-fitting statistical models problem, according to the recommendation of a previous study [33,34]. Maternal age was categorized into two groups: 30 years or younger and older than age 30. Sibling status was categorized as only child vs. child with sibling at the time of the first survey. (Note: as information on the child’s sibling status was assessed only during the first survey round, the status might have changed later if a child was born in the household after the first survey round.) Wealth index was measured with wealth status and living environment, including housing quality, water and sanitation quality, and access to energy sources, and then constructed by principal component analysis from three equally weighted components – a housing quality index, a services quality index, and a consumer durables index – with ranges from 0 to 1 [35]. Subsequently, these values were ranked into wealth quintiles from the poorest to the wealthiest. The selection of these variables as potential confounders was guided by a review of the previous literature.

### Statistical methods

We performed multilevel multivariable logistic regression with a random intercept model to examine the association between maternal literacy and completion of four kinds of child vaccination while accounting for the clustering of observations at the community level for each country in 2006. To test for a moderating effect of state-run health centers and community health workers on that association, cross-level interaction terms were constructed by multiplying two dummy variables representing presence of a state-run health center or community health workers and maternal literacy.

First, models 1–1 and 1–2 considered only individual-level and household-level variables with and without controlling for paternal literacy. Then, the two community-level variables were included as explanatory variables into separate regression models (model 2–1 for state-run health center and model 3–1 for community health worker). Finally, we extended models 2–1 and 3–1 by adding the cross-level interaction terms to test the moderating effect of two community-level variables on the association between maternal literacy and completion of four kinds of vaccinations (models 2–2 and 3–2). Analyses were carried out in 2016 using SAS version 9.3 [36].

## Results

Table 1 shows descriptive characteristics of the child samples by individual-, household-, and community-level variables. More than 60% of mothers in Ethiopia and India were illiterate. The percentage of mothers aged 30 or younger was much higher in India than in Ethiopia (79.2% vs. 49.4%). Only 27.3% of respondents in Ethiopia and 15.6% of respondents in India had had state-run health centers in their community 4 years prior to the survey. The percentage of respondents living in a community where community health workers wer present 4 years prior to the survey was 63.5% in Ethiopia and 51.2% in India.

**Table 1.** Descriptive characteristics of child samples across Ethiopia and India in 2006.

		Ethiopia		India	
Variable	Categories	Total: N(%)	Completed 4 vaccinations: N(%)	Total: N(%)	Completed 4 vaccinations: N(%)
Maternal literacy	Illiterate	740 (64.0)	636 (85.9)	885 (60.8)	841 (95.0)
	Literate	417 (36.0)	397 (95.2)	570 (39.2)	543 (95.2)
Paternal literacy	Illiterate	485 (41.9)	406 (83.7)	572 (39.3)	541 (94.6)
	Literate	672 (58.1)	627 (93.3)	883 (60.7)	843 (95.5)
Maternal age	30 ≦	572 (49.4)	514 (89.9)	1152 (79.2)	1097 (95.2)
	30 >	585 (50.6)	519 (88.7)	303 (20.8)	287 (94.7)
Gender of child	Male	617 (53.3)	555 (90.0)	770 (52.9)	728 (94.5)
	Female	540 (46.7)	478 (88.5)	685 (47.1)	656 (95.8)
Sibling status*	With Sibling	865 (74.8)	762 (88.1)	639 (43.9)	612 (95.8)
	Only child	292 (25.2)	271 (92.8)	816 (56.1)	772 (94.6)
Wealth	Poorest	225 (19.4)	208 (92.4)	309 (19.3)	282 (91.3)
	Poor	221 (19.1)	204 (92.3)	288 (19.8)	271 (94.1)
	Middle	234 (20.2)	206 (88.0)	287 (19.7)	281 (97.9)
	Wealthy	237 (20.5)	207 (87.3)	293 (20.1)	284 (96.9)
	Wealthiest	240 (20.7)	208 (86.7)	278 (19.1)	266 (95.7)
Type of residence	Rural	733 (63.4)	644 (87.9)	362 (75.1)	320 (88.4)
	Urban	424 (36.6)	389 (91.7)	1093 (24.9)	1064 (97.3)
Region	(Ethiopia)				
	Addis Ababa	198 (17.1)	195 (98.5)		
	Amhara	272 (23.5)	250 (91.9)		
	Oromia regional state	220 (19.0)	192 (87.3)		
	Southern Nations, Nationalities, and Peoples’ region	312 (27.0)	242 (77.6)		
	Tigray	155 (13.4)	154 (99.4)		
	(India)				
	Coastal Andhra			492 (33.8)	472 (95.9)
	Rayalaseema			459 (31.6)	450 (98.0)
	Telangana			504 (34.6)	462 (95.9)
Existence of a state-run health center in community	No	841 (72.7)	761 (90.5)	1228 (84.4)	1161 (94.5)
	Yes	316 (27.3)	272 (86.1)	227 (15.6)	223 (98.2)
Existence of community	No	423 (36.6)	363 (85.8)	710 (48.8)	664 (93.5)
health workers in community	Yes	734 (63.4)	670 (91.3)	745 (51.2)	720 (96.6)
Total		1,157 (100)		1,455 (100)	

*At the time of the first round of the survey.

The percentage of children who completed the four kinds of vaccinations according to the categories of individual-level and community-level variables showed mixed results by country. In India, the percentage was similar for both literate and illiterate parents, while it was higher among literate parents than illiterate ones in Ethiopia. The percentage was higher in communities without a state-run health center than in communities with a center in Ethiopia, whereas the opposite pattern appeared in India.

The results from adjusted multivariable multilevel analyses for Ethiopia and India are presented in Tables 2 and 3, respectively. When individual- and household-level covariates were adjusted for, maternal literacy was associated with vaccination status only in Ethiopia. Specifically, literate mothers in Ethiopia were 2.54 times (95% Confidence Interval (CI): 1.35–4.75, p = 0.004) more likely to complete all four kinds of vaccinations for their child as compared to illiterate mothers (model 1–1 in Table 2). This relationshiremained robust even after adjusting for paternal literacy (Adjusted Odds Radio (AOR) = 2.27, 95% CI: 1.18–4.39, p < 0.01 in Ethiopia; model 1–2 in Table 2). The presence of a state-run health center in the respondent’s community was positively associated with child vaccination status only in India. That is, children living in a communities where a state-run health center was located were 6.6 times (95% CI: 1.57–27.70, p < 0.01) more likely to have received the four vaccinations than children in communities without a state-run health center (model 2–1 in Table 3). The presence of community health workers was not directly associated with child vaccination status in either country.

**Table 2.** Results from multilevel logistic models of the association between maternal literacy and child completion of four vaccinations in Ethiopia.

		Adjusted odds ratios (95% Confidence interval)					
	Null	1–1^a^	1–2^b^	2–1^c^	2–2^d^	3–1^e^	3–2^f^
**Fixed effect**							
Maternal literacy (ref = illiterate)							
Literate		**2.54 (1.35–4.75)	*2.27 (1.18–4.39)	*2.19 (1.13–4.24)	*2.36 (1.07–5.20)	*2.30 (1.19–4.47)	**4.84 (1.75–13.36)
Paternal literacy (ref = illiterate)							
Literate			1.32 (0.81–2.13)	1.31 (0.81–2.12)	1.30 (0.80–2.11)	1.31 (0.81–2.12)	1.28 (0.79–2.08)
Presence of state-run health center (ref = none)							
Yes				0.69 (0.30–1.61)	0.74 (0.30–1.83)		
Maternal l﻿iteracy * Presencexistence of a state-run health center					0.80 (0.23–2.79)		
Presence of community health workers (ref = none)							
Yes						1.34 (0.58–3.12)	1.80 (0.72–4.46)
Maternal literacy * Presence of community health workers							*0.29 (0.09–0.96)
**Random effect**							
Community variation (Standard error)	1.378 (0.575)	0.282 (0.199)	0.285 (0.199)	0.284 (0.216)	0.295 (0.221)	0.299 (0.212)	0.331 (0.222)
Intraclass correlation	29.5	7.9	8.0	7.9	8.2	8.3	9.1

^a^Model 1–1: adjusted for only maternal literacy.

^b^Model 1–2: added paternal literacy to model 1–1.

^c^Model 2–1: added presence of health center to model 1–2.

^d^Model 2–2: added the interaction term of maternal literacy and presence of health center to model 2–1.

^e^Model 3–1 : added presence of health workers to model 1–2.

^f^Model 3–2: added the interaction term of maternal literacy and presence of health workers to model 3–1. ► All models were additionallyadjusted for maternal age, gender of child, sibling status, wealth level, type of residence, and region. *p < 0.05; **p < 0.01; ***p < 0.001; ****p < 0.0001

**Table 3.** Results from multilevel logistic models of the association between maternal literacy and child completion of four vaccinations in India.

		Adjusted odds ratios (95% Confidence interval)					
	Null	1–1^a^	1–2^b^	2–1^c^	2–2^d^	3–1^e^	3–2^f^
**Fixed effect**							
Maternal literacy (ref = illiterate)							
Literate		1.84 (0.96–3.51)	1.73 (0.89–3.39)	1.67 (0.85–3.28)	1.46 (0.73–2.90)	1.74 (0.89–3.41)	^†^2.26 (0.98–5.19)
Paternal literacy (ref = illiterate)							
Literate			1.24 (0.67–2.29)	1.22 (0.66–2.24)	1.24 (0.68–2.29)	1.24 (0.67–2.29)	1.25 (0.68–2.30)
Presence of a state-run health center (ref = none)							
Yes				**6.60 (1.57–27.70)	*3.09 (0.62–15.38)		
Maternal literacy * Presence of a state-run health center					5.55 (0.48–63.62)		
Presence of community health workers (ref = none)							
Yes						1.07 (0.43–2.63)	1.33 (0.49–3.58)
Maternal literacy * Presence of community health workers							0.51 (0.15–1.76)
**Random effect**							
Community variation (Standard error)	1.655 (0.470)	1.176 (0.242)	1.159 (0.421)	0.784 (0.378)	0.765 (0.374)	1.204 (0.437)	1.227 (0.442)
Intraclass correlation	33.5	26.3	26.0	19.2	18.9	26.8	27.2

^a^Model 1–1: adjusted for only maternal literacy.

^b^Model 1–2: added paternal literacy to model 1–1.

^c^Model 2–1: added presence of health center to model 1–2.

^d^Model 2–2: added the interaction term of maternal literacy and presence of health center to model 2–1.

^e^Model 3–1 : added presence of health workers to model 1–2.

^f^Model 3–2: added the interaction term of maternal literacy and presence of health workers to model 3–1. ► All models were additionally adjusted for maternal age, gender of child, sibling status, wealth level, type of residence, and region. *p < 0.05; **p < 0.01; ***p < 0.001; ****p < 0.0001.

**Figure 1.**
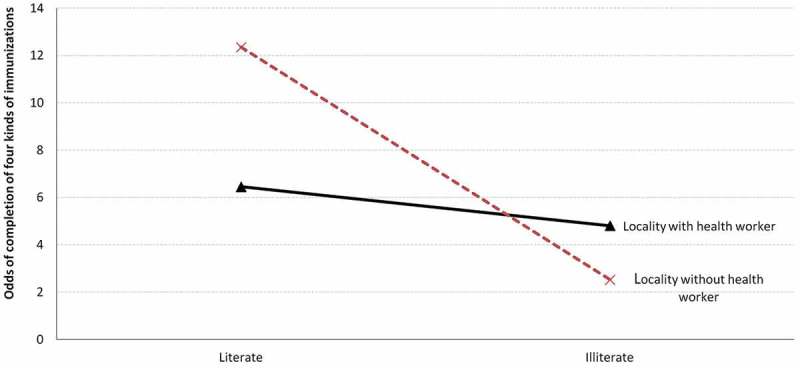
Differential effect of maternal literacy on the child immunization status according to the presence of community health workers in Ethiopia.

The cross-level interaction effect between presence of a state-run health center and maternal literacy was not significant in either country, indicating that having a state-run health center in the respondent’s community did not moderate the association between maternal literacy and child vaccinations (model 2–2 in Tables 2 and 3). However, the cross-level interaction between community health workers and maternal literacy was statistically significant in Ethiopia (model 3–2 in Table 2). This result indicates that the gap in the odds of completing the four kinds of vaccinations between illiterate and literate mothers was smaller in communities with community health workers than in communities without community health workers (Figure 1).

Random effect results revealed that about 29.5% and 33.5% of the total variation in child receipt of these four vaccines in Ethiopia and India, respectively, was associated with clustering of the outcome within communities. Generally, variation in the outcome at the community level decreased as we accounted for more explanatory variables.

Tables S1 and S2 provide the estimates for the relationships between the other explanatory variables and the outcome in Ethiopia and India. For example, the odds of completing all four kinds of vaccinations were far lower for children living in rural areas as compared to children living in urban areas in India. In Ethiopia, the odds of getting all four kinds of vaccinations for children living in the Oromia regional state and Southern Nations, Nationalities, and Peoples’ Region were much lower compared to children living in Addis Ababa.

## Discussion

The two hypotheses examined in our study were that (a) maternal literacy has a positive association with child vaccination status after adjusting for potential confounding factors, and (b) presence of a state-run health center or community health workers in the community would reduce the gap in vaccination status of children between literate and illiterate mothers. Results from multilevel logistic regression analyses supported the first hypothesis only in Ethiopia. The finding that illiterate mothers in Ethiopia are less likely to finish all four kinds of vaccinations for their child compared to literate mothers was consistent with results from previous studies. In general, the link between maternal illiteracy and poor child health-related outcomes is stronger in LMICs [15,17–19,37–39] than in high-income countries [40–42]. Prior research has found that literate mothers tend to be more likely to be knowledgeable about target diseases, vaccination schedules, or benefits of vaccination, which may motivate them to get their children fully immunized, whereas illiterate mothers are less likely to be exposed to this information [43]. In addition, although social support may buffer the adverse effect of illiteracy on health outcomes [20], the illiterate might have limited social supports due to weak social networks [13,44–46], which makes them more alienated from essential vaccination knowledge.

This study found evidence that the presence of a state-run health center seemed beneficial for child vaccination only in India. In addition, there was no evidence that a state-health center in the community contributed to reducing the gap in the rate of full vaccination status between children from illiterate mothers and children from literate mothers in either country. The direct association result is similar to a prior finding that proximity to a health center is positively associated with child vaccination in India [47]. However, another study in India found that availability of health infrastructure in a community had only a modest effect on vaccination coverage in a rural area [26].

These mostly null findings about this form of health infrastructure may be evidence that presence of a state-run health center alone does not capture the challenges associated with making health centers effective. First, distrust in the quality of health centers, or cultural factors, may hinder a mother’s visit to a health center. Second, the presence of state-run health centers in the community does not guarantee their proper functioning. For example, some state-run health centers are not equipped with the cold chain system for maintaining vaccines at proper temperature due to unstable and inadequate financial resources [48]. Therefore, these health centers are open for vaccination service only for a few days when the vaccine is delivered to the health center, resulting in a lack of service days for vaccination [49–51]. Third, a lack of transportation or the cost of transportation to such centers also can prevent community members from utilizing vaccination services through a state-run health center [52]. Finally, if mothers living in communities without a state-run health center can easily access a private clinic, financially and geographically, then the presence of a state-run health center in the community may not be associated with child vaccination status because mothers in both types of communities could ostensibly be getting their children vaccinated elsewhere. Thus, variation in the effect of a state-run health center on vaccination status may arise from variation in these conditions in each country.

Lastly, our analysis offered evidence that the presence of community health workers may act as a moderator on the relationship between maternal illiteracy and child vaccination status in Ethiopia. That is, the potential harmful effect of maternal illiteracy on child vaccination status might be more pronounced among the children living in areas without community health workers in Ethiopia. Community health workers are responsible not only for administering vaccines but also for monitoring vaccination coverage and conducting door-to-door visits for all children to be immunized in a timely way by disseminating information on vaccination and identifying and mobilizing the target group [26,47,53]. Face-to-face and one-on-one interpersonal communication with community health workers is considered to be an effective means for reducing barriers in access to information for marginalized populations such as children of illiterate mothers [25,54].

As with state-run health centers, however, the presence of community health workers does not ensure that the system is properly functioning. Although AWW started their service in 1975 as front-line workers of Integrated Child Development Services [55], the community health worker system has been insufficient in number and/or lacks capacity in terms of knowledge, skills, attitudes or motivation [56,57]. In addition, organizational support such as resources, remuneration or incentive, trainings, and working conditions is always lacking [58–60]. This lack of resources might explain, in part, a non-significant direct association between community health workers and child vaccination status in India. To address challenges with their community health worker system, India launched the National Rural Health Mission in 2005 to rejuvenate its rural health system. The country ambitiously trained a cadre of 250,000 community health workers and provided them with a monetary incentive and allowance for covering difficult areas with the goal to increase child vaccination rates [61].

The majority of children who fail to receive a completed vaccination schedule are mainly from marginalized groups, including children with illiterate parents [14,15]. Our findings may have the encouraging implication that well-functioning community health workers can contribute to increasing access to child vaccination services for children with illiterate parents.

### Limitations

A few limitations of the current study need to be acknowledged. First, the data for the study are more than 10 years old. Thus, the situation might have changed regarding illiteracy rates or the quality of health workers or health centers in Ethiopia and India. However, a change in those factors does not harm the validity of this study’s interpretation that properly functioning health workers may improve vaccination rates among children with illiterate mothers. In addition, the results of our paper may offer lessons to other LMICs that are currently going through similar situations to what Ethiopia and India experienced 10 years ago. Second, information on the vaccination status of the child was collected via respondents’ recall when respondents did not have a vaccination card, which can raise the possibility of ‘recall bias’. However, the prevalence of each type of vaccination in our study sample was compatible with figures provided by international agencies such as WHO/UNICEF [62], thus suggesting that recall bias was not a major issue in our data. Third, the variables for community health workers and state-run health centers in this study only represent the presence of those community sources, and do not reflect quality. Lastly, data from India were only from Andhra Pradesh and Telangana. Therefore, results may not be generalizable to other parts of the country. Finally, the cross-sectional study design limits inferences on the directional causality of the associations found in this study.

## Conclusion

Generally, health infrastructure such as public health centers and health human resources such as community health workers are important for vaccination. The results of our analyses showed that the availability of community health workers may reduce the impact of maternal illiteracy on child vaccination status, although the result depended on the country. This finding emphasizes the potential importance of implementing a high-quality community health worker system in expanding vaccination coverage among underserved children in LMICs, especially in countries with a high rate of illiteracy. Although reducing illiteracy itself is a more fundamental and ideal solution, this goal would take a long time to achieve. Thus, improving the number and quality of community health workers may be a good additional step in improving vaccination rates among children of illiterate mothers.

## Data Availability

Data are available from the UK Data Service website (at https://discover.ukdataservice.ac.uk/series/?sn=2000060). Users are required to register and apply for a password with the UK Data Service and sign a confidentiality agreement before obtaining access to the data. Also, users are asked to inform the UK Data Service and Young Lives of analysis or publication resulting from their work with the data set.

## Paper context

Child immunization coverage in LMICs is still far from complete, mainly among marginalized people such as children with illiterate mothers. This novel study examines whether state-run health centers and community health workers play a moderating role in the association between maternal literacy and child immunization. Community health workers reduced the gap in child immunization between illiterate and literate mothers in Ethiopia.
