# Supplementary material for: Association between maternal literacy and child vaccination in Ethiopia and southeastern India and the moderating role of health workers: a multilevel regression analysis of the Young Lives study
Source: Glob Health Action. 2019 Apr 8;12(1):1581467. doi: 10.1080/16549716.2019.1581467 (PMC6461100; doi:10.1080/16549716.2019.1581467)
Supplement: Supplemental Material [file ZGHA_A_1581467_SM8710.docx]

Table S1. Full results from multilevel logistic models on the association between maternal literacy and child completion of four vaccinations in Ethiopia

|  | **Adjusted Odds-Ratios** | | | | | | | | |
| --- | --- | --- | --- | --- | --- | --- | --- | --- | --- |
|  | 1-1^a^ | 1-2^b^ | | 2-1^c^ | | 2-2^d^ | | 3-1^e^ | 3-2^f^ |
| Maternal literacy (Ref=illiterate) | | | | | | | | | |
| Literate | ^**^2.54  (1.35-4.75) | ^*^2.27  (1.18-4.39) | | ^*^2.19  (1.13-4.24) | | ^*^2.36  (1.07-5.20) | | ^*^2.30  (1.19-4.47) | ^**^4.84  (1.75-13.36) |
| Paternal literacy (Ref=illiterate) | | | | | | | | | |
| Literate |  | 1.32  (0.81-2.13) | | 1.31  0.81-2.12) | | 1.30  (0.80-2.11) | | 1.31  (0.81-2.12) | 1.28  (0.79-2.08) |
| Existence of state-run health center (Ref= none) | | | | | | | | | |
| Yes |  |  | | 0.69  (0.30-1.61) | | 0.74  (0.30-1.83) | |  |  |
| Maternal Literacy * existence of a state-run health center | |  | |  | | 0.80  (0.23-2.79) | |  |  |
| Existence of community health worker (Ref=none) | | | | | | | | | |
| Yes | | |  | |  | | 1.34  (0.58-3.12) | | 1.80  (0.72-4.46) |
| Maternal literacy * existence of community health workers | | |  | |  | |  | | ^*^0.29  (0.09-0.96) |
| Age of caregiver (Ref= ≤30) | | | | | | | | | |
| > 30 | 1.04  (0.67-1.60) | 1.05  (0.68-1.63) | | 1.05  (0.68-1.63) | | 1.05  (0.68-1.62) | | 1.04  (0.67-1.61) | 1.04  (0.67-1.62) |
| Gender of child (Ref=male) | | | | | | | | | |
| Female | 0.90  (0.60-1.34) | 0.90  (0.60-1.34) | | 0.90  (0.60-1.35) | | 0.90  (0.61-1.35) | | 0.90  (0.60-1.34) | 0.90  (0.60-1.35) |
| Sibling status ^§^ (Ref=with siblings) | | | | | | | | | |
| Only child | 1.33  (0.76-2.34) | 1.32  (0.75-2.32) | | 1.32  (0.75-2.32) | | 1.31  (0.74-2.31) | | 1.31  (0.74-2.30) | 1.27  (0.72-2.24) |
| Wealth level (Ref=poorest) |  |  | |  | |  | |  |  |
| Poor | 1.15  (0.64-2.06) | 1.11  (0.62-2.01) | | 1.09  (0.60-1.98) | | 1.09  (0.60-1.98) | | 1.13  (0.62-2.04) | 1.17  (0.65-2.12) |
| Middle | 0.92  (0.49-1.72) | 0.89  (0.47-1.67) | | 0.87  (0.46-1.64) | | 0.87  (0.46-1.64) | | 0.91  (0.48-1.71) | 0.95  (0.50-1.80) |
| Wealthy | 1.39  (0.62-3.10) | 1.33  (0.59-2.98) | | 1.28  (0.57-2.89) | | 1.29  (0.57-2.91) | | 1.38  (0.61-3.12) | 1.43  (0.62-3.26) |
| Wealthiest | 1.21  (0.48-3.04) | 1.12  (0.44-2.85) | | 1.07  (0.42-2.73) | | 1.07  (0.42-2.73) | | 1.17  (0.46-2.98) | 1.13  (0.44-2.90) |
| Type of residency (Ref=urban) |  |  | |  | |  | |  |  |
| Rural | 1.19  (0.49-2.89) | 1.19  (0.49-2.90) | | 1.07  (0.43-2.70) | | 1.09  (0.43-2.78) | | 1.14  (0.46-2.84) | 1.22  (0.47-3.13) |
| Region (Ref=Addis Ababa) | | | | | |  | |  |  |
| Amhara | 0.30  (0.06-1.43) | 0.31  (0.07-1.45) | | 0.30  (0.06-1.43) | | 0.29  (0.06-1.40) | | 0.27  (0.05-1.36) | 0.27  (0.05-1.35) |
| Oromia | ^*^0.19  (0.04-0.89) | ^*^0.20  (0.04-0.96) | | ^*^0.17  (0.04-0.89) | | ^*^0.16  (0.03-0.85) | | ^*^0.21  (0.04-1.01) | ^†^0.22  (0.04-1.09) |
| Southern Nations, Nationalities, and Peoples’ region | ^***^0.09  (0.02-0.38) | ^**^0.10  (0.02-0.41) | | ^**^0.09  (0.02-0.38) | | ^**^0.09  (0.02-0.39) | | ^**^0.10  (0.02-0.44) | ^**^0.09  (0.02-0.41) |
| Tigray | 4.72  (0.36-61.94) | 4.80  (0.36-63.19) | | 4.03  (0.36-61.94) | | 3.89  (0.28-54.10) | | 4.40  (0.32-58.58) | 3.82  (0.27-53.33) |

^a^ Model 1-1: adjusted for only maternal literacy / ^b^Model 1-2: added paternal literacy to the model 1-1 / ^c^ Model 2-1: added existence of health center to the model 1-2/ ^d^ Model 2-2: added the interaction term of maternal literacy and existence of health center to the model 2-1/ ^e^ Model 3-1 : added existence of health worker to the model 1-2 / ^f^ Model 3-2: added the interaction term of maternal literacy and existence of health worker to the model 3-1

^§^ : at the time of the first survey

^*^p <0.05; ^**^p<0.01; ^***^p<0.001; ^† :^ marginally significant

Table S2. Full results from multilevel logistic models on the association between maternal literacy and child completion of four vaccinations in India.

|  | **Adjusted Odds-Ratios** | | | | | | |
| --- | --- | --- | --- | --- | --- | --- | --- |
|  | 1-1^a^ | 1-2^b^ | 2-1^c^ | | 2-2^d^ | 3-1^e^ | 3-2^f^ |
| Maternal literacy (Ref=illiterate) |  | | | | | | |
| Literate | 1.84  (0.96-3.51) | 1.73  (0.89-3.39) | 1.67  (0.85-3.28) | | 1.46  (0.73-2.90) | 1.74  (0.89-3.41) | ^†^2.26  (0.98-5.19) |
| Paternal literacy (Ref=illiterate) |  | | | | | | |
| Literate |  | 1.24  (0.67-2.29) | 1.22  (0.66-2.24) | | 1.24  (0.68-2.29) | 1.24  (0.67-2.29) | 1.25  (0.68-2.30) |
| Existence of state-run health center (Ref=none) |  | | | | | | |
| Yes |  |  | ^**^6.60  (1.57-27.70) | | ^*^3.09  (0.62-15.38) |  |  |
| Maternal Literacy * existence of a state-run health center |  |  |  | | 5.55  (0.48-63.62) |  |  |
| Existence of community health worker (Ref=none) |  |  |  | |  |  |  |
| Yes |  |  |  | |  | 1.07  (0.43-2.63) | 1.33  (0.49-3.58) |
| Maternal literacy * existence of community health workers |  |  |  | |  |  | 0.51  (0.15-1.76) |
| Age of caregiver (Ref= ≤30) |  | | |  | | | |
| > 30 | 0.98  (0.52-1.83) | 0.97  (0.52-1.82) | 0.99  (0.53-1.85) | | 0.99  (0.53-1.86) | 0.97  (0.52-1.82) | 0.95  (0.50-1.78) |
| Gender of child (Ref=male) |  | | | | | | |
| Female | 1.24  (0.73-2.10) | 1.24  (0.73-2.11) | 1.24  (0.73-2.10) | | 1.24  (0.73-2.11) | 1.24  (0.73-2.11) | 1.25  (0.74-2.13) |
| Sibling status^§^ (Ref=with siblings) | | | | | | | |
| Only child | 1.11  (0.64-1.91) | 1.12  (0.65-1.93) | 1.12  (0.65-1.93) | | 1.10  (0.64-1.90) | 1.12  (0.65-1.91) | 1.15  (0.66-1.99) |
| Wealth level (Ref=poorest) |  |  |  | |  |  |  |
| Poor | 1.28  (0.50-3.27) | 1.23  (0.48-3.17) | 1.27  (0.50-3.25) | | 1.29  (0.51-3.29) | 1.24  (0.48-3.19) | 1.27  (0.49-3.29) |
| Middle | 1.57  (0.54-4.58) | 1.49  (0.51-4.40) | 1.50  (0.52-4.36) | | 1.54  (0.53-4.46) | 1.49  (0.50-4.40) | 1.53  (0.52-4.54) |
| Wealthy | 1.64  (0.56-4.58) | 1.56  (0.52-4.65) | 1.63  (0.55-4.84) | | 1.62  (0.55-4.78) | 1.55  (0.52-4.65) | 1.60  (0.53-4.80) |
| Wealthiest | 1.58  (0.49-5.13) | 1.44  (0.43-4.82) | 1.49  (0.45-4.93) | | 1.45  (0.44-4.76) | 1.44  (0.43-4.82) | 1.42  (0.43-4.74) |
| Living area (Ref=urban) |  |  |  | |  |  |  |
| Rural | ^**^0.19  (0.06-0.58) | ^**^0.19  (0.06-0.58) | ^***^0.11  (0.04-0.35) | | ^***^0.12  (0.04-0.36) | ^**^0.19  (0.06-0.61) | ^**^0.18  (0.06-0.58) |
| Region (Ref=Coastal Andhra**)** |  |  |  | |  |  |  |
| Rayalaseema | 1.42  (0.43-4.72) | 1.40  (0.42-4.65) | 1.24  (0.49-3.89) | | 1.24  (0.40-3.87) | 1.42  (0.41-4.85) | 1.45  (0.42-5.01) |
| Telangana | 0.50  (0.19-1.28) | 0.50  (0.19-1.28) | 0.44  (0.18-1.06) | | 0.43  (0.18-1.03) | 0.51  (0.19-1.38) | 0.55  (0.20-1.50) |

^a^ Model 1-1: adjusted for only maternal literacy / ^b^Model 1-2: added paternal literacy to the model 1-1 / ^c^ Model 2-1: added existence of health center to the model 1-2/ ^d^ Model 2-2: added the interaction term of maternal literacy and existence of health center to the model 2-1/ ^e^ Model 3-1 : added existence of health worker to the model 1-2 / ^f^ Model 3-2: added the interaction term of maternal literacy and existence of health worker to the model 3-1

^§^ : at the time of the first survey

^*^p <0.05; ^**^p<0.01; ^***^p<0.001; ^† :^ marginally significant
